# Supplementary material for: CD8 T cell epitope generation toward the continually mutating SARS-CoV-2 spike protein in genetically diverse human population: Implications for disease control and prevention
Source: PLoS One. 2020 Dec 10;15(12):e0239566. doi: 10.1371/journal.pone.0239566 (PMC7728258; doi:10.1371/journal.pone.0239566)
Supplement: S1 Fig — (PDF) [file pone.0239566.s003.pdf]

S1 Fig. Multiple sequence alignment of 1000 S protein sequences with reference S protein of SARS-CoV-2 (QHD43416.1) showing position 601 to 720 that contains D614G mutation with high frequency

```
QHD43416.1  601   GTNTSNQVAVLYQDVNCTEVPVAIHADQLTPTWRVYSTGSNVFQTRAGCLIGAEHVNNSYECDIPIGAGICASYQTQTNSPRRARSVASQSIIAYTMSLGAENSVAYSNNSIAIPTNFTI  720
QJF75467.1  601   ........................................................................................................................  720
QII57278.1  601   ........................................................................................................................  720
QJR85953.1  601   ........................................................................................................................  720
QJX45031.1  601   ........................................................................................................................  720
QJS39567.1  601   ........................................................................................................................  720
QJF77846.1  601   ........................................................................................................................  720
QJR87129.1  601   ........................................................................................................................  720
QIZ16509.1  601   ........................................................................................................................  720
QIS60546.1  601   ........................................................................................................................  720
QJD20632.1  601   ........................................................................................................................  720
QJS39579.1  601   ........................................................................................................................  720
QLC91196.1  601   ........................................................................................................................  720
QIZ16559.1  601   ........................................................................................................................  720
QKV38208.1  601   ........................................................................................................................  720
QJT73034.1  601   ........................................................................................................................  720
QIU81585.1  601   ........................................................................................................................  720
QKV39923.1  601   ........................................................................................................................  720
QJG65956.1  601   ........................................................................................................................  720
QIS30425.1  601   ........................................................................................................................  720
QKS90791.1  601   ........................................................................................................................  720
QJQ84843.1  601   ........................................................................................................................  720
QIZ97039.1  601   ........................................................................................................................  720
QKU32813.1  601   ........................................................................................................................  720
QKV41471.1  601   ........................................................................................................................  720
QIS60489.1  601   ........................................................................................................................  720
QJE38426.1  601   ........................................................................................................................  720
QLC48052.1  601   ........................................................................................................................  720
QII87830.1  601   ......................................................Y.................................................................  720
QHU79173.2  601   ........................................................................................................................  720
QIS61422.1  601   ........................................................................................................................  720
QJS53398.1  601   ........................................................................................................................  720
QKU28894.1  601   ........................................................................................................................  720
QIO04367.1  601   ........................................................................................................................  720
QIT07011.1  601   ........................................................................................................................  720
QII57268.2  601   ........................................................................................................................  720
QIS61254.1  601   ........................................................................................................................  720
QJE38822.1  601   .......................................................................................................X................  720
QKG89546.1  601   ........................................................................................................................  720
QIU80913.1  601   ........................................................................................................................  720
QKK14611.1  601   ...................................................................................T....................................  720
QKV06271.1  601   ........................................................................................................................  720
QKV06859.1  601   ........................................................................................................................  720
QIU81885.1  601   ........................................................................................................................  720
QIU81873.2  601   ....................................................V...................................................................  720
QJW39952.1  601   ........................................................................................................................  720
QIS30165.1  601   ........................................................................................................................  720
QIA98583.1  601   ........................................................................................................................  720
QKV35819.1  601   ........................................................................................................................  720
QJS57147.1  601   .....................A..................................................................................................  720
QJF76007.1  601   .......................................................................................................L................  720
QJC19455.1  601   ........................................................................................................................  720
QKU31481.1  601   ........................................................................................................................  720
QIS60906.1  601   ........................................................................................................................  720
QJR84837.1  601   .......................................X................................................................................  720
QKU33257.1  601   ........................................................................................................................  720
QJQ84676.1  601   ........................................................................................................................  720
QIV65033.1  601   ........................................................................................................................  720
QKU28906.1  601   ........................................................................................................................  720
QJR84993.1  601   ........................................................................................................................  720
QIS30335.1  601   ........................................................................................................................  720
QIU80973.1  601   ........................................................................................................................  720
QJQ39524.1  601   ........................................................................................................................  720
QJG65957.1  601   ........................................................................................................................  720
QJR84873.1  601   ........................................................................................................................  720
QKY78084.1  601   ........................................................................................................................  720
QLB39236.1  601   ........................................................................................................................  720
QJR84969.1  601   .................................................................X......................................................  720
QJX45333.1  601   ........................................................................................................................  720
QIK50427.1  601   .............G..........................................................................................................  720
QIS61338.1  601   ........................................................................................................................  720
QJC20993.1  601   ........................................................................................................................  720
QIS61468.1  601   ........................................................................................................................  720
QIS30105.1  601   .............X..........................................................................................................  720
QKV06235.1  601   ........................................................................................................................  720
QJS54814.1  601   ........................................................................................................................  720
QIA20044.1  601   ........................................................................................................................  720
QJF11959.1  601   ........................................................................................................................  720
QJD47202.1  601   ........................................................................................................................  720
QJR85701.1  601   ........................................................................................................................  720
QKS65788.1  601   ........................................................................................................................  720
QIZ13143.1  601   ........................................................................................................................  720
QLC94832.1  601   ........................................................................................................................  720
QKS89927.1  601   ........................................................................................................................  720
QJR87477.1  601   ............................................................................H...........................................  720
QJR86013.1  601   ........................................................................................................................  720
QKU53313.1  601   ........................................................................................................................  720
QKV07519.1  601   ........................................................................................................................  720
QKY74810.1  601   ........................................................................................................................  720
QJD48279.1  601   ........................................................................................................................  720
QJR94041.1  601   ........................................................................................................................  720
QJR85833.1  601   ........................................................................................................................  720
QKV38653.1  601   ........................................................................................................................  720
QJQ38816.1  601   ........................................................................................................................  720
QJR88113.1  601   ........................................................................................................................  720
QJR84585.1  601   ........................................................................................................................  720
QJR88761.1  601   ........................................................................................................................  720
QJG65951.1  601   ........................................................................................................................  720
QIZ13861.1  601   ........................................................................................................................  720
QIZ13789.1  601   .............G.......I..................................................................................................  720
QKE46016.1  601   ........................................................................................................................  720
QIC53204.1  601   ........................................................................................................................  720
QJR84957.1  601   ........................................................................................................................  720
QKV25357.1  601   ........................................................................................................................  720
```

```
QIJ96493.1  601  ........................................................................................................................  720
QIZ15717.1  601  ........................................................................................................................  720
QKS65584.1  601  ........................................................................................................................  720
QIQ08810.1  601  ........................................................................................................................  720
QJX44586.1  601  .............G..........................................................................................................  720
QHR84449.1  601  ........................................................................................................................  720
QIZ14569.1  601  ........................................................................................................................  720
QKG89150.1  601  .............G..........................................................................................................  720
QIS60930.1  601  ........................................................................................................................  720
QKV39263.1  601  .............G..........................................................................................................  720
QKV37344.1  601  ........................................................................................................................  720
QLC47285.1  601  .............G..........................................................................................................  720
QJT72350.1  601  ........................................................................................................................  720
QJR85857.1  601  ........................................................................................................................  720
QJR85965.1  601  ........................................................................................................................  720
QJR84537.1  601  ........................................................................................................................  720
QJQ27878.1  601  ........................................................................................................................  720
QIQ50092.1  601  ........................................................................................................................  720
QKT21158.1  601  ........................................................................................................................  720
QJA17276.1  601  .............G..........................................................................................................  720
QIV15164.1  601  ...........................................X............................................................................  720
QJS54754.1  601  .............G..........................................................................................................  720
QJQ28105.1  601  .............G..........................................................................................................  720
QKI36829.1  601  ........................................................................................................................  720
QKG88142.1  601  .............G..........................................................................................................  720
QKM76822.1  601  ........................................................................................................................  720
QKW95051.1  601  .............G.........................A................................................................................  720
QKS90131.1  601  ........................................................................................................................  720
QIS60978.1  601  ........................................................................................................................  720
QKU31265.1  601  ........................................................................................................................  720
QJX45356.1  601  .............G..........................................................................................................  720
QJF11971.1  601  ......................................................X.................................................................  720
QJT73010.1  601  .............G..........................................................................................................  720
QKU54045.1  601  ........................................................................................................................  720
QIZ13299.1  601  ........................................................................................................................  720
QJT43608.1  601  .............G..........................................................................................................  720
QKV06079.1  601  .............G..........................................................................................................  720
QIZ64624.1  601  ........................................................................................................................  720
QKS66532.1  601  .............G..........................................................................................................  720
QKV42778.1  601  .............G..........................................................................................................  720
QKS65656.1  601  ........................................................................................................................  720
QIS60582.1  601  ........................................................................................................................  720
QJC20391.1  601  .............G..........................................................................................................  720
QKU32237.1  601  ........................................................................................................................  720
QIZ13179.1  601  ........................................................................................................................  720
QKV40847.1  601  .............G..........................................................................................................  720
QKG90434.1  601  .............G..........................................................................................................  720
QKV06175.1  601  ........................................................................................................................  720
QJT43584.1  601  .............G..........................................................................................................  720
QKN19803.1  601  .............G..........................................................................................................  720
QJT72086.1  601  ........................................................................................................................  720
QJE38606.1  601  .............G..........................................................................................................  720
QLC47069.1  601  ........................................................................................................................  720
QJX44574.1  601  .............G..........................................................................................................  720
QHZ00379.1  601  ........................................................................................................................  720
QIU78825.1  601  ........................................................................................................................  720
QKJ68364.1  601  .............G..........................................................................................................  720
QKE45704.1  601  ........................................................................................................................  720
QLC46385.1  601  .............G..........................................................................................................  720
QLC47117.1  601  .............G..........................................................................................................  720
QJX68694.1  601  .............G..........................................................................................................  720
QKG91082.1  601  .............G..........................................................................................................  720
QLA47775.1  601  .............G..........................................................................................................  720
QKG88694.1  601  .............G..........................................................................................................  720
QJQ04352.1  601  .............G.............................................................S............................................  720
QKV39455.1  601  .............G..........................................................................................................  720
QJA42177.1  601  .............G..........................................................................................................  720
QKU31085.1  601  ........................................................................................................................  720
QJU11589.1  601  .............X..........................................................................................................  720
QKY65277.1  601  .............G..........................................................................................................  720
QJT72242.1  601  .............G..........................................................................................................  720
QJR88221.1  601  .............G..........................................................................................................  720
QJY51864.1  601  .............G..........................................................................................................  720
QJX70300.1  601  .............G......S...................................................................................................  720
QJR87789.1  601  .............G...........V..............................................................................................  720
QJX68978.1  601  .............G......................................................................................V...................  720
QKV35783.1  601  .............G..........................................................................................................  720
QKG86966.1  601  .............G..........................................................................................................  720
QJR87261.1  601  .............G..........................................................................................................  720
QKE45498.1  601  .............G..............................X...........................................................................  720
QKV27551.1  601  .............G..........................................................................................................  720
QJY39929.1  601  ........................................................................................................................  720
QLC47908.1  601  .............G..........................................................................................................  720
QLC92852.1  601  .............G..........................................................................................................  720
QKU32141.1  601  .............G..........................................................................................................  720
QKO00486.1  601  .............G..........................................................................................................  720
QJX69795.1  601  .............G..........................................................................................................  720
QJS53410.1  601  .............G..........................................................................................................  720
QKU30389.1  601  .............G..........................................................................................................  720
QJD23524.1  601  ........................................................................................................................  720
QKN20643.1  601  .............G..........................................................................................................  720
QJR88005.1  601  .............G..........................................................................................................  720
QJZ28114.1  601  .............G......................................V...................................................................  720
QJG65949.1  601  ........................................................................................................................  720
QKG90614.1  601  .............G..........................................................................................................  720
QKE45932.1  601  .............G.......................................Z..................................................................  720
QKE45656.1  601  ........................................................................................................................  720
QKE44665.1  601  .............G.........................................................V................................................  720
QKN20847.1  601  .............G..........................................................................................................  720
QIZ16197.1  601  .............G..........................................................................................................  720
QJQ38972.1  601  .............G..........................................................................................................  720
QKE45824.1  601  ........................................................................................................................  720
QKU37093.1  601  .............G.....................................................................V....................................  720
QKU30629.1  601  .............G..........................................................................................................  720
QKN19611.1  601  .............G..........................................................................................................  720
QKV39024.1  601  ........................................................................................................................  720
QKU31397.1  601  ........................................................................................................................  720
QJT43572.1  601  .............G..........................................................................................................  720
QJD25529.1  601  .............G................S.........................................................................................  720
QJX69218.1  601  ...X.........G..........................................................................................................  720
QKV37031.1  601  .............G..........................................................................................................  720
QJC19491.1  601  .............G..........................................................................................................  720
QJR84909.1  601  ......................XX................................................................................................  720
QJD47718.1  601  ........................................................................................................................  720
```

QJC20043.1 601 ....G.. 720
QIQ49882.1 601 720
QLA09870.1 601 ....G.. 720
QKU31901.1 601 ....G.. 720
QJQ84640.1 601 720
QLC48016.1 601 ....G.. 720
QJI53955.1 601 ....G.. 720
QKK12815.1 601 ....G.. 720
QKR85081.1 601 ....G.. 720
QKV05911.1 601 720
QJA17468.1 601 ....G.. 720
QIZ64470.1 601 ....G.. 720
QJD47358.1 601 ....G.. 720
QJD25085.1 601 ....G.. 720
QJA17596.1 601 ....G.. 720
QJF77194.1 601 ....G.. 720
QKG86990.1 601 ....G.. 720
QKQ63616.1 601 .............G...S.................................... 720
QJS54898.1 601 ....G.. 720
QKU31205.1 601 ....G.. 720
QJQ83944.1 601 ....G.. 720
QJD24377.1 601 ....G.. 720
QJT72278.1 601 ..........F..G.. 720
QKG89918.1 601 ....G.. 720
QKV38964.1 601 ....G.. 720
QKV40775.1 601 ....G.. 720
QKE61804.1 601 ....G.. 720
QJF76438.1 601 ....G.. 720
QKN19515.1 601 .............G...L...................... 720
QIZ64530.1 601 ....G.. 720
QKY77964.1 601 ....X.. 720
QJR89217.1 601 ....G.. 720
QJR86037.1 601 ....G.. 720
QKY60121.1 601 ....G.. 720
QKW89191.1 601 ....G.. 720
QKS91031.1 601 720
QJX69482.1 601 ....G.. 720
QKG88946.1 601 ....G.. 720
QJS57267.1 601 ....G.. 720
QJT43452.1 601 ....G.. 720
QKU32081.1 601 .............G........S... 720
QJS56895.1 601 ....G.. 720
QJS53578.1 601 ....G.. 720
QKU31133.1 601 .............G.........................F.. 720
QJZ32209.1 601 ....G.. 720
QIS30295.1 601 ....G.. 720
QJC20367.1 601 ....G.. 720
QJX44430.1 601 ....G.. 720
QIZ97051.1 601 ....G.. 720
QKU32465.1 601 ....G.. 720
QJD47442.1 601 ....G.. 720
QJS39543.1 601 720
QJR87909.1 601 ....G.. 720
QJW69235.1 601 ....G.. 720
QJU70329.1 601 ....G.. 720
QKV06379.1 601 ....G.. 720
QJU70545.1 601 ....G.. 720
QJQ82996.1 601 ....G.. 720
QIZ64578.1 601 ....G.. 720
QKE45692.1 601 .............G...F............ 720
QJR94641.1 601 ....G.. 720
QKR84321.1 601 ....G.. 720
QIZ15969.1 601 ....G.. 720
QKU32045.1 601 ....G.. 720
QJX70519.1 601 ....G.. 720
QJS39507.1 601 ....G.. 720
QJX44634.1 601 .............G...S.............. 720
QJR84369.1 601 ....G.. 720
QIX13999.1 601 .............G..H... 720
QJS57135.1 601 ....G.. 720
QKE45884.1 601 .............G........X... 720
QJX68786.1 601 ....G.. 720
QLC91688.1 601 720
QKG81727.1 601 ....G.. 720
QJH92167.1 601 .............G..H... 720
QLB38609.1 601 ....G.. 720
QJA17632.1 601 ....G.. 720
QKG89462.1 601 ....G.. 720
QKN19755.1 601 ....G.. 720
QLB39021.1 601 ....G.. 720
QJF75443.1 601 ....G.. 720
QJR85569.1 601 ....G.. 720
QKE53933.1 601 .............G..X... 720
QIS30615.1 601 ....G.. 720
QJS39627.1 601 ....G.. 720
QKM76630.1 601 ....G.. 720
QKU53553.1 601 720
QJW00291.1 601 ....G.. 720
QJS54106.1 601 ....G.. 720
QKV35267.1 601 ....G.. 720
QIZ15645.1 601 ....G.. 720
QKU37621.1 601 ....G.. 720
QJA17192.1 601 ....G.. 720
QJX45007.1 601 ....G.. 720
QJT43704.1 601 ....G.. 720
QJR87465.1 601 ....G.. 720
QJA17524.1 601 ....X.. 720
QJR88497.1 601 ....G.. 720
QKE50930.1 601 ....G.. 720
QKU32765.1 601 ....G.. 720
QKU33389.1 601 ....G.. 720
QJX68658.1 601 ....G.. 720
QKJ68497.1 601 ....HG.. 720
QIZ13765.1 601 ....GF... 720
QKU31721.1 601 ....G.. 720
QKX47945.1 601 ....G.. 720
QJD23273.1 601 ....G.. 720
QJR86313.1 601 ....G.. 720
QJF75779.1 601 ....G.. 720
QLC91064.1 601 ....G.. 720
QIS30115.1 601 ....G.. 720
QJR87957.1 601 ....G.. 720
QJX68418.1 601 ....G.. 720
QJR93333.1 601 ....G.. 720

QKU32429.1	601	............G...	720
QKJ68737.1	601	............G...	720
QIZ15585.1	601	............G...	720
QJX69134.1	601	............G...	720
QJG65954.1	601	....	720
QKO25674.1	601	............G...	720
QJS53386.1	601	............G...	720
QKN19911.1	601	............G...	720
QKI28601.1	601	............G...	720
QKV38988.1	601	............G...	720
QJU70245.1	601	............G...	720
QKO25794.1	601	............G...	720
QJY40061.1	601	....	720
BCG67531.1	601	............G...	720
QKV40583.1	601	............G...	720
QJD47800.1	601	............G...	720
QKE50894.1	601	............G...	720
QIU78719.1	601	............G...	720
QKV35279.1	601	............G...	720
QJR87897.1	601	............G...L.................	720
QKE61684.1	601	............G...	720
QJR87321.1	601	....	720
QKN61217.1	601	....W.......................................	720
QJX70327.1	601	............G...	720
QJX70251.1	601	............G...	720
QKO25770.1	601	............G...	720
QKU31445.1	601	............G...	720
QKG86786.1	601	............G...	720
QJQ27854.1	601	............G...	720
QKG89558.1	601	............G...	720
QKV38532.1	601	............G...	720
QLA47679.1	601	............G...	720
QJR84729.1	601	..................XX..	720
QKX47933.1	601	............G...	720
QKU30329.1	601	............G...	720
QJC20343.1	601	............G...	720
QKN20943.1	601	............G...	720
QKC05236.1	601	............G...	720
QKV25225.1	601	............X...	720
QKN19995.1	601	............G...	720
BCG44664.1	601	............G...	720
QJY39881.1	601	............G...	720
QJQ83680.1	601	............G...	720
QKX47969.1	601	............G...	720
QJD48543.1	601	....	720
QLC47920.1	601	............G...	720
QKV37536.1	601	............G...	720
QJR94065.1	601	............G...	720
QKU31121.1	601	............G...	720
QKG81475.1	601	............G...	720
QJF75335.1	601	............G...	720
QJX45307.1	601	............G...	720
QKV37584.1	601	............G...	720
QKU37501.1	601	............G...	720
QJS54286.1	601	............G...	720
QJG65952.1	601	....	720
QKN19551.1	601	............G...	720
QJR86781.1	601	............G...	720
QJG65958.1	601	....	720
QKQ63760.1	601	............G...	720
QJY78056.1	601	............G...	720
QKE51026.1	601	............G...	720
QJR93153.1	601	............G..X..	720
QJX44562.1	601	............G...	720
QKJ68605.1	601	............G...	720
QIZ64685.1	601	............G..H..	720
QKX47981.1	601	............G...	720
QKU53445.1	601	....	720
QJE38546.1	601	............G..L..	720
QJZ27921.1	601	............G...X.......................................	720
QJT72806.1	601	............G...	720
QLC47597.1	601	............G...	720
QJQ84364.1	601	............G...	720
QJI53943.1	601	............G...	720
QKV49386.1	601	....Q.......................................	720
QKO24139.1	601	............G...	720
QJF76906.1	601	............G...	720
QJF76750.1	600	....	719
QJS57039.1	601	............G...	720
QKN20763.1	601	............G...	720
QKX46299.1	601	............G...L.......................	720
QKU37885.1	601	............G...	720
QLC47896.1	601	............G.......F...	720
QJY40469.1	601	............G...	720
QJI07354.1	601	............G...	720
QJZ28347.1	601	............G...	720
QKV42838.1	601	............G...	720
QJY40289.1	601	....	720
QKM76906.1	601	....	720
QJF11812.1	601	............G...	720
QKG91034.1	601	............G...	720
QIU80949.1	601	....	720
QJD48915.1	601	............G...	720
QKU31505.1	601	....	720
QJR90981.1	601	....	720
QJR84441.1	601	............G...	720
QLA10140.1	601	............G...	720
QKG90986.1	601	............G...	720
QKU32273.1	601	............G...	720
QKG86870.1	601	............G...	720
QJR86997.1	601	....	720
QKX65028.1	601	............G...	720
QJQ83764.1	601	............G...	720
QKR84357.1	601	............G.........................X...	720
QJD23609.1	601	............G...	720
QKS89843.1	601	....	720
QJY40517.1	601	............G...	720
QJZ28203.1	601	............G...	720
QJX45344.1	601	............G...	720
QJU11577.1	601	............X...	720
QJQ84280.1	601	............G...	720
QJT72614.1	601	............G...I...	720
QLC46853.1	601	............G...	720
QJC19935.1	601	............G...	720

```
QJQ84736.1  601   .............G..........................................................................................................  720
QJY40565.1  601   .............G..........................................................................................................  720
QKG89366.1  601   .............G..........................................................................................................  720
QKX65052.1  601   .............G..........................................................................................................  720
QKE44809.1  601   .............G..........................................................................................................  720
QKU37261.1  601   .............G..........................................................................................................  720
QIZ13131.1  601   ........................................................................................................................  720
QKW89143.1  601   .............G................S.........................................................................................  720
QJC19851.1  601   .............G..........................................................................................................  720
QJI54407.1  601   .............G..........................................................................................................  720
QJD25193.1  601   .............G..........................................................................................................  720
QJR86577.1  601   ........................................................................................................................  720
QKV09211.1  601   ........................................................................................................................  720
QJQ84292.1  601   .............G..........................................................................................................  720
QIZ13407.1  601   .............G..........................................................................................................  720
QKU31229.1  601   .............G..........................................................................................................  720
QJE39085.1  601   ..........................................................XXX...........................................................  720
QKR84285.1  601   .............G..............................................................H...........................................  720
QKN19623.1  601   .............G...........................X..............................................................................  720
QKU37177.1  601   .............G..........................................................................................................  720
QKX65004.1  601   .............G.........................X................................................................................  720
QJX69519.1  601   .............G..........................................................................................................  720
QJC20271.1  601   .............G..........................................................................................................  720
QJQ82972.1  601   .............G..........................................................................................................  720
QJR84429.1  601   .............G...........................................................................................S..............  720
QJS53506.1  601   .............G..........................................................................................................  720
QKU31769.1  601   .............G..........................................................................................................  720
QIZ13969.1  601   .............G..........................................................................................................  720
QJQ83668.1  601   .............G..........................................................................................................  720
QJR86289.1  601   ........................................................................................................................  720
QLC46253.1  601   .............G..........................................................................................................  720
QKV37332.1  601   .............G..........................................................................................................  720
QJD47094.1  601   .............G.....................................................XX...................................................  720
QJR87693.1  601   .............G..........................................................................................................  720
QJR84393.1  601   ........................................................................................................................  720
QJR89421.1  601   .............G..........................................................................................................  720
QKF95522.1  601   .................................................................................Q......................................  720
QKV38905.1  601   .............G..........................................................................................................  720
QJR91413.1  601   ........................................................................................................................  720
QKU32561.1  601   .............G..........X...............................................................................................  720
QJR87501.1  601   .............G......S...................................................................................................  720
QJT72386.1  601   .............G..........................................................................................................  720
QKU32057.1  601   .............G..........................................................................................................  720
QKG27877.1  601   .............G..........................................................................................................  720
QKG87470.1  601   .............G..........................................................................................................  720
QKM76402.1  601   .............G..........................................................................................................  720
QKY59893.1  600   .............G..........................................................................................................  719
QJW39940.1  601   ..................................................................................X.....................................  720
QJY78152.1  601   .............G..........................................................................................................  720
QKV37632.1  601   .............G.........................................................................................L................  720
QLB39177.1  601   .............G..........................................................................................................  720
QKU31097.1  601   .............G..........................................................................................................  720
QJR93441.1  601   ........................................................................................................................  720
QKR84333.1  601   .............G.........................X................................................................................  720
QKG89534.1  601   .............G..........................................................................................................  720
QJX68490.1  601   .............G..........................................................................................................  720
QIS30455.1  601   .............G..........................................................................................................  720
QJY40217.1  601   .............G..........................................................................................................  720
QHS34546.1  600   ........................................................................................................................  719
QKU37405.1  601   .............G..X.......................................................................................................  720
QKG64075.1  601   .............G..........................................................................................................  720
QJR94881.1  601   .............G..........................................................................................................  720
QIU81429.1  601   ........................................................................................................................  720
QKU31985.1  601   .............G.........................................................X................................................  720
QKX65076.1  601   .............G..........................................................................................................  720
QKU37429.1  601   ........................................................................................................................  720
QKE11078.1  601   .............X..........................................................................................................  720
QKV37428.1  601   .............G..........................................................................................................  720
QJR90309.1  601   .............G..........................................................................................................  720
QKJ68545.1  601   .............G..........................................................................................................  720
QJF77206.1  601   .............G..........................................................................................................  720
QJR91617.1  601   .............G..........................................................................................................  720
QKE45680.1  601   .............G..........................................................................................................  720
QJR88437.1  601   .............G..........................................................................................................  720
QJU70569.1  601   .............G...................................................................W......................................  720
QIS61170.1  601   .............G..........................................................................................................  720
QJD47118.1  601   .............G..........................................................................................................  720
QJR93057.1  601   ........................................................................................................................  720
QKU33413.1  601   ........................................................................................................................  720
QKU37081.1  601   .............G..........................................................................................................  720
QJS54922.1  601   .............G..........................................................................................................  720
QJR94353.1  601   .............G..........................................................................................................  720
QJQ39692.1  601   .............G..........................................................................................................  720
QJR93465.1  601   .............G..........................................................................................................  720
QKE45668.1  601   .............G..........................................................................................................  720
QIZ14797.1  601   ........................................................................................................................  720
QKV42586.1  601   .............G..........................................................................................................  720
QJC20487.1  601   ........................................................................................................................  720
QKV37392.1  601   ........................................................................................................................  720
QKU33209.1  601   .............G..........................................................................................................  720
QKV39443.1  601   ........................................................................................................................  720
QJQ39788.1  601   .............G..........................................................................................................  720
QJQ83140.1  601   .............G..........................................................................................................  720
QJQ84220.1  601   .............G..........................................................................................................  720
QJA16794.1  601   .............G..........................................................................................................  720
QKU37645.1  601   .............G...........X...................................X..........................................................  720
QJR88317.1  601   .............G..........................................................................................................  720
QKV37296.1  601   .............G..........................................................................................................  720
QIS30575.1  601   .............G..........................................................................................................  720
QIQ49832.1  601   ........................................................................................................................  720
QJD23141.1  601   .............G..........................................................................................................  720
QJI54323.1  601   ........................................................................................................................  720
QJG65947.1  601   ........................................................................................................................  720
QJR85989.1  601   ........................................................................................................................  720
QKV37368.1  601   .............G..........................................................................................................  720
QJY40457.1  601   ........................................................................................................................  720
QKX64992.1  600   .............G..........................................................................................................  719
QKV25105.1  601   .............G..........................................................................................................  720
QJR89457.1  601   .............G..........................................................................................................  720
QKU31973.1  601   .............G.....................................................................................................XX...  720
QLA46612.1  601   ........................................................................................................................  720
QKR85033.1  601   .............G..........................................................................................................  720
QJR94593.1  601   ........................................................................................................................  720
QKG90626.1  601   .............G..........................................................................................................  720
```

QKG89750.1 601 720
QJF75455.1 601 ....G.. 720
QJR95097.1 601 720
QJD47130.1 601 ....G.. 720
QJA16640.1 601 ....G.. 720
QJS54742.1 597 716
QKU31961.1 601 ....G.. 720
QKR86269.1 601 ....G.. 720
QKV37308.1 601 720
QKE49364.1 601 720
QKV37284.1 601 ....G.. 720
QKU53985.1 601 720
QJR88737.1 601 ....G.. 720
QJR89181.1 601 ....G.. 720
QJI54098.1 601 720
QJD47658.1 601 ....G.. 720
QJR90753.1 601 ....X.. 720
QJQ39944.1 601 ....G.. 720
QJR84561.1 601 ....XXXXXX... 720
QIS30525.1 601 720
QJW00459.1 601 ....G.. 720
QKV37236.1 601 ....G.. 720
QKE44989.1 601 ....XXXXXX... 720
QLC47621.1 601 ....G.. 720
QKV38725.1 601 ....G.. 720
QKR84745.1 601 ....G.. 720
QKX65088.1 601 ....G.. 720
QLC47704.1 601 ....G.. 720
QKX65100.1 601 .............G..H... 720
QLC46361.1 601 720
QJR93357.1 601 720
QKR84420.1 601 ....X.. 720
QJQ39068.1 601 ....G.. 720
QKR84697.1 601 ....G.. 720
QKE46004.1 601 ....XXXXXX... 720
QJD49071.1 601 720
QKG87086.1 601 ....G.. 720
QJQ39812.1 601 ....G.. 720
QJR85497.1 601 ....G.. 720
QJY51780.1 601 ....G.. 720
QKE49172.1 601 720
QKU28426.1 601 720
QJS57231.1 601 720
QJW69139.1 601 ....G.. 720
QJZ28359.1 601 ....G.. 720
QJR89229.1 601 ....G.. 720
QJA16508.1 601 ....G.. 720
QKV38028.1 601 ....G.. 720
QJF76414.1 601 ....G.. 720
QJR89157.1 601 ....G.. 720
QJR84945.1 601 ....XXXXXXX.. 720
QKE45740.1 601 ....XXXXXXXX.. 720
QKV35843.1 601 ....G.. 720
QJQ82804.1 601 ....G.. 720
QKG88730.1 601 ....G.. 720
QKG91130.1 601 720
QJF76163.1 601 ....H....................................... 720
QJR89337.1 601 .............G..X................... 720
QJQ38624.1 601 ....X... 720
QJR88425.1 601 ....G.. 720
QJR93081.1 601 ....G.. 720
QKV37464.1 601 ....G.. 720
QJC20415.1 601 720
QKV37452.1 601 720
QKU54477.1 601 ....G.. 720
QJD48099.1 601 720
QKE45025.1 601 ....G.. 720
QJX69507.1 601 ....G.. 720
QJQ83164.1 601 ....XXXXXXXXXX.. 720
QJR93561.1 601 .............G..........X... 720
QKU37477.1 601 ....XXXXXXXXXX... 720
QIQ50102.1 601 720
QKU53709.1 601 ....X.. 720
QJD48759.1 601 720
QJR89313.1 601 ....X.. 720
QKV38761.1 601 ....G.. 720
QKV38052.1 601 ....G.. 720
QKV38520.1 601 720
QJR85905.1 601 720
QJR85677.1 601 ....G.. 720
QJX45271.1 601 ....G.. 720
QKS91043.1 601 720
QJF74903.1 601 ....G.. 720
QJR90297.1 601 ....G.. 720
QIU81213.1 601 ....G.. 720
QKV38544.1 601 ....G.. 720
QJF75419.1 601 ....G.. 720
QKE45037.1 601 ....G.. 720
QJX44490.1 589 ....G.. 708
QLB39320.1 601 ....G.. 720
QJX44454.1 588 ....G.. 707
QIZ14917.1 601 720
QKR86449.1 601 ....G.. 720
QJX69627.1 601 ....G.. 720
QKN19575.1 601 ....G.. 720
QJC19995.1 601 ....G.. 720
QKV37512.1 601 ....G.. 720
QIS60558.1 601 720
QJE39109.1 601 720
QKV39252.1 601 720
QJR95349.1 601 ....G.. 720
QJR87645.1 601 ....G.. 720
QKS67443.1 601 ....----------................................ 710
QIS30645.1 601 720
QJD25442.1 588 ....G.. 707
QKR84541.1 601 720
QJR91905.1 601 720
QIZ13069.1 601 ....G.. 720
QJQ39032.1 601 ....G.. 720
QKS90047.1 601 720
QJY51804.1 601 ....G.. 720
QKS89975.1 601 720
QJR89277.1 601 ....G.. 720
QKV07399.1 601 720

```
QJR84345.1  601  ........................................................................................................................  720
QJI54335.1  601  ........................................................................................................................  720
QKV37572.1  601  ........................................................................................................................  720
QJF76534.1  601  .............G..........................................................................................................  720
QKV37908.1  601  .............G..........................................................................................................  720
QJC19863.1  601  ........................................................................................................................  720
QKV38617.1  601  ........................................................................................................................  720
QJQ83512.1  601  .............G..........................................................................................................  720
QJR86733.1  601  .............G..........................................................................................................  720
QLC47165.1  601  .............G..........................................................................................................  720
QJX70119.1  601  .............G..........................................................................................................  720
QJR91353.1  601  .............G..........................................................................................................  720
QJD24617.1  601  .............G..........................................................................................................  720
QLB39105.1  601  ........................................................................................................................  720
QIS30225.1  601  .............G..........................................................................................................  720
QKV25801.1  601  .............G..........................................................................................................  720
QJA16868.1  601  ........................................................................................................................  720
QKV06403.1  601  ........................................................................................................................  720
QJR95361.1  601  .............G..........................................................................................................  720
QIQ49902.1  601  ........................................................................................................................  720
QKE49074.1  601  .............G..........................................................................................................  720
QJR89361.1  601  ...............................................X........................................................................  720
QKG89570.1  601  .............G..........................................................................................................  720
QJQ38804.1  601  ........................................................................................................................  720
QJR95109.1  601  .............G..........................................................................................................  720
QJR87945.1  601  ...............................................X........................................................................  720
QKV39839.1  601  .............G..........................................................................................................  720
QJR87777.1  601  .............G..........................................................................................................  720
QKR84637.1  601  ...............................................X........................................................................  720
QIZ13569.1  601  .............G..........................................................................................................  720
QKV38677.1  601  ........................................................................................................................  720
QJR93297.1  601  .............G..........................................................................................................  720
QJR87489.1  601  .............G..........................................................................................................  720
QJR89397.1  601  .............G.................................X........................................................................  720
QJR84657.1  601  ...................XXXXXXXXXXXXXXX......................................................................................  720
QKV35735.1  601  .............G..........................................................................................................  720
QJR85977.1  601  ........................................................................................................................  720
QJR84753.1  601  ...................XXXX.................................................................................................  720
QJD24099.1  601  .............G..........................................................................................................  720
QJD47586.1  601  .............G..........................................................................................................  720
QJR87633.1  601  ........................................................................................................................  720
QKG87818.1  601  .............G..........................................................................................................  720
QJR91281.1  601  ...............................................X........................................................................  720
QJR87081.1  601  .............G..........................................................................................................  720
QJA16580.1  601  .............G..........................................................................................................  720
QJR86829.1  601  ........................................................................................................................  720
QKV38833.1  601  ........................................................................................................................  720
QJR91257.1  601  .............G..........................................................................................................  720
QJD25349.1  601  .............G..........................................................................................................  720
QJQ38996.1  601  .............G..........................................................................................................  720
QJR86625.1  601  .............G..........................................................................................................  720
QKV25165.1  601  .............G..........................................................................................................  720
QJD23153.1  601  .............G..........................................................................................................  720
QJR94617.1  601  ........................................................................................................................  720
QKK14100.1  601  .............G..................................................................................................XXXXXXXX  720
QKR84805.1  601  .............G..........................................................................................................  720
QJR93801.1  601  ........................................................................................................................  720
QIS61374.1  601  ........................................................................................................................  720
QJR90345.1  601  .............G..........................................................................................................  720
QJE39097.1  601  ........................................................................................................................  720
QJR86937.1  601  ........................................................................................................................  720
QKG88322.1  601  .............G..........................................................................................................  720
QJR95301.1  601  ........................................................................................................................  720
QJR90501.1  601  .............G..........................................................................................................  720
QIS60822.1  601  .............G..........................................................................................................  720
QKN61229.1  592  ..........................................................................-----.........................................  706
QJI54086.1  601  ........................................................................................................................  720
QJD24809.1  601  .............G..........................................................................................................  720
QJF76474.1  601  .............G..........................................................................................................  720
QKV05983.1  601  .XX..........X..........................................................................................................  720
QJD47214.1  601  .............G..........................................................................................................  720
QKR84925.1  601  .............G............................................................H.............................................  720
QKU54537.1  593  ........................................................................................................................  712
QKS89867.1  601  ........................................................................................................................  720
QKK14335.1  601  .............G..........................................................................................................  720
QJR84549.1  601  ........................................................................................................................  720
QJR93861.1  601  .............G..........................................................................................................  720
QJF77050.1  601  .............G..........................................................................................................  720
QKR84841.1  601  .............G..........................................................................................................  720
QKV06355.1  601  ........................................................................................................................  720
QIV65022.1  601  ........................................................................................................................  720
QJX68586.1  601  .............G........................................X................................................................  720
QJR90717.1  601  ........................................................................................................................  720
QJD47478.1  601  .............G..........................................................................................................  720
QJR90465.1  601  .............G..........................................................................................................  720
QJI54229.1  601  ........................................................................................................................  720
QJQ39440.1  601  .............G..........................................................................................................  720
QJF76990.1  601  .............G..........................................................................................................  720
QLC47681.1  601  .............G..........................................................................................................  720
QKU53721.1  601  ........................................................................................................................  720
QJF75239.1  601  .............G..........................................................................................................  720
QKR86605.1  601  ........................................................................................................................  720
QKG88970.1  601  .............G..........................................................................................................  720
QJR86589.1  601  .............G..........................................................................................................  720
QJR94953.1  601  ........................................................................................................................  720
QJE39038.1  601  ........................................................................................................................  720
QJS54766.1  601  .............G............XXXX...............X..........................................................................  720
QJZ32221.1  601  .............G..........................................................................................................  720
QIZ14437.1  601  .............G..........................................................................................................  720
QJR84705.1  601  ........................................................................................................................  720
QKF96007.1  574  .............G..........................................................................................................  693
QIS61528.1  600  ........................................................................................................................  719
QKV37728.1  601  ........................................................................................................................  720
QKV38568.1  601  ........................................................................................................................  720
QJR94257.1  601  .............G..........................................................................................................  720
QKS89831.1  601  ........................................................................................................................  720
QKV37752.1  601  .............G..........................................................................................................  720
QKR84889.1  601  ........................................................................................................................  720
QLC46721.1  601  .............G..........................................................................................................  720
QJR87177.1  601  .............G..........................................................................................................  720
QKY77928.1  601  ........................................................................................................................  720
QKV40427.1  601  .............G..........................................................................................................  720
QKU28486.1  601  ........................................................................................................................  720
QLC47800.1  601  .............G..........................................................................................................  720
```

```
QJR94497.1  601   .............G..........................................................................................................  720
QKG87146.1  601   .............G..........................................................................................................  720
QKG87794.1  601   .............G..........................................................................................................  720
QKV37704.1  601   .............G..........................................................................................................  720
QJD47310.1  601   .............G..........................................................................................................  720
QJR91317.1  601   ........................................................................................................................  720
QKR85153.1  601   ........................................................................................................................  720
QJI54383.1  601   ........................................................................................................................  720
QJR94725.1  601   .............G..........................................................................................................  720
QKR85069.1  601   ........................................................................................................................  720
QJD47298.1  601   .............G..........................................................................................................  720
QKG90230.1  601   .............G..........................................................................................................  720
QIZ14353.1  601   .............G..........................................................................................................  720
QIQ49922.1  601   ........................................................................................................................  720
QIS30135.1  601   ........................................................................................................................  720
QJR94929.1  601   .............G..........................................................................................................  720
QIV65044.1  601   .............G..........................................................................................................  720
QKV25873.1  601   .............G..........................................................................................................  720
QLC47848.1  601   .............G..........................................................................................................  720
QJR91641.1  601   ........................................................................................................................  720
QLC46949.1  601   .............G..........................................................................................................  720
QKK14233.1  601   .............G.......................................XXXXXXXXXXXXXXXXXXXXXXXX...........................................  720
QKG90722.1  601   .............G..........................................................................................................  720
QKU53301.1  601   ........................................................................................................................  720
QJD48507.1  601   ........................................................................................................................  720
QJR89385.1  601   .............G..........................................................................................................  720
QLC47465.1  601   .............G..........................................................................................................  720
QJR92001.1  601   .............G..........................................................................................................  720
QLC46157.1  601   .............G..........................................................................................................  720
QKR84997.1  601   .............G..........................................................................................................  720
QKU53229.1  601   ........................................................................................................................  720
QJD23249.1  601   ........................................................................................................................  720
QKV25129.1  601   ........................................................................................................................  720
QJD24315.1  601   ........................................................................................................................  720
QJR89937.1  601   .............G..........................................................................................................  720
QJA17180.1  601   .............G..........................................................................................................  720
QKU53385.1  601   ........................................................................................................................  720
QJR86721.1  601   ........................................................................................................................  720
QKV38040.1  601   .............G..........................................................................................................  720
QKG87050.1  601   .............G..........................................................................................................  720
QJD23907.1  601   .............G..........................................................................................................  720
QJI54122.1  601   ........................................................................................................................  720
QJR90681.1  601   .............G..........................................................................................................  720
QJR92985.1  601   .............G..........................................................................................................  720
QJD23379.1  601   .............G..........................................................................................................  720
QKU52869.1  601   .........................................................................................................XXXXX..........  720
QKR84649.1  601   .............G..........................................................................................................  720
QKE44749.1  601   .............G..........................................................................................................  720
QJR87249.1  601   .............G..........................................................................................................  720
QKN20583.1  566   .............G..........................................................................................................  685
QJR92637.1  601   .............G..........................................................................................................  720
QJR89445.1  601   .............G.................................X....X...............................................X...................  720
QJE39121.1  601   ........................................................................................................................  720
QJR94569.1  601   .............G..........................................................................................................  720
QJI54371.1  601   ........................................................................................................................  720
QJR90513.1  601   .............G..........................................................................................................  720
QJR86109.1  601   .............G..........................................................................................................  720
QLC46241.1  601   .............G..........................................................................................................  720
QKG87638.1  601   ........................................................................................................................  720
QJR93873.1  601   .............G..........................................................................................................  720
QJF74963.1  601   .............G..........................................................................................................  720
QKV38785.1  601   ........................................................................................................................  720
QKV37692.1  601   ........................................................................................................................  720
QIZ13647.1  601   .............G..........................................................................................................  720
QJR94305.1  601   .............G..........................................................................................................  720
QJR93417.1  601   ........................................................................................................................  720
QLC46961.1  601   .............G..........................................................................................................  720
QKY60189.1  601   .............G..........................................................................................................  720
QKR85429.1  601   .............G..........................................................................................................  720
QIZ14195.1  601   .............G..........................................................................................................  720
QJD23775.1  601   .............G..........................................................................................................  720
QKK14185.1  601   .............G..................................................................................................XXXXXXXX  720
QJE38978.1  601   ........................................................................................................................  720
QKK14076.1  601   .............G.................................................................................................XXXXXXXXX  720
QKU52845.1  601   .............G..........................................................................................................  720
QKG89666.1  601   .............G................................V.........................................................................  720
QKE12094.1  601   .............G................................................................................................XXXXXXXXXX  720
QKV25429.1  561   ........................................................................................................................  680
QJC20714.1  601   .............G..........................................................................................................  720
QJS54730.1  601   .............G..........................................................................................................  720
QKY60177.1  601   .............G..........................................................................................................  720
QKG87806.1  601   .............G..........................................................................................................  720
QKG88838.1  601   .............G..........................................................................................................  720
QLC47776.1  561   .............G..........................................................................................................  680
QJZ32186.1  601   ........................................................................................................................  720
QKS89903.1  601   ........................................................................................................................  720
QJR94809.1  601   .............G..........................................................................................................  720
QKR85609.1  601   .............G..........................................................................................................  720
QJR84849.1  601   ...........................................................................XXXXXXXXXXXXX................................  720
QKV35447.1  601   .............G..........................................................................................................  720
QJF74891.1  601   .............G..........................................................................................................  720
QKU31313.1  601   ........................................................................................................................  720
QJR90321.1  601   .............G..........................................................................................................  720
QKV08263.1  601   ........................................................................................................................  720
QJR88929.1  601   ........................................................................................................................  720
QJD23283.1  601   .............G..........................................................................................................  720
QKR86245.1  601   .............G..........................................................................................................  720
QKV42946.1  601   .............G..........................................................................................................  720
QJR93237.1  601   .............G..........................................................................................................  720
QJY51648.1  601   .............G..........................................................................................................  720
QJR91029.1  601   .............G..........................................................................................................  720
QIS60606.1  601   ........................................................................................................................  720
QJX68394.1  601   .............G..........................................................................................................  720
QLC46709.1  601   .............G..........................................................................................................  720
QJR94893.1  601   ........................................................................................................................  720
QIU81657.1  601   .............G..........................................................................................................  720
QKR85309.1  601   .............G..........................................................................................................  720
QJR86601.1  601   .............G..........................................................................................................  720
QKV38857.1  601   ........................................................................................................................  720
QKU32297.1  601   .....XXXXXXXXXXXXXXX....................................................................................................  720
QKK14112.1  601   .............G.................................................................................................XXXXXXXXX  720
QJC20475.1  601   .............G..........................................................................................................  720
QJR90561.1  601   .............G..........................................................................................................  720
QJZ32129.1  601   .............G..........................................................................................................  720
```

```
QJD48819.1  601   ........................................................................................................................  720
QJR86877.1  601   .............G..........................................................................................................  720
QJR89205.1  601   .............G..........................................................................................................  720
QJA17432.1  601   .............G..........................................................................................................  720
QIU81369.1  601   .............G..........................................................................................................  720
QIS60654.1  601   .............G..........................................................................................................  720
QIZ13895.1  601   ........................................................................................................................  720
QKR85237.1  601   .............G..........................................................................................................  720
QKU53733.1  601   ........................................................................................................................  720
QJR94221.1  601   ........................................................................................................................  720
QIZ14833.1  601   .............G..........................................................................................................  720
QJR91149.1  601   .............G..........................................................................................................  720
QKR84661.1  601   ........................................................................................................................  720
QKV37740.1  601   ........................................................................................................................  720
QKR86185.1  601   .............G..........................................................................................................  720
QKV67308.1  601   ........................................................................................................................  720
QKV08047.1  601   .............G..........................................................................................................  720
QKR84961.1  601   ........................................................................................................................  720
QIS30595.1  601   ........................................................................................................................  720
QKG87014.1  601   .............G..........................................................................................................  720
QJR94377.1  601   .............G..........................................................................................................  720
QJC20259.1  601   .............G..........................................................................................................  720
QJR86661.1  601   .............G..........................................................................................................  720
QJF77278.1  601   .............G..........................................................................................................  720
QKR85093.1  601   .............G..........................................................................................................  720
QJR93141.1  601   ........................................................................................................................  720
QKV37944.1  601   .............G..........................................................................................................  720
QKR85177.1  601   .............G............................................................................X.............................  720
QJD47610.1  601   .............G..........................................................................................................  720
QJR91737.1  601   .............G..........................................................................................................  720
QKU32789.1  601   ........................................................................................................................  720
QKR85249.1  601   .............G..........................................................................................................  720
QKU52833.1  601   ........................................................................................................................  720
QKV38773.1  601   .....XXXXXXXXXXXXXXXXXXXXXXXXXXXXX.......................T..............................................................  720
QJC19887.1  601   .............G..........................................................................................................  720
QKV37224.1  601   .............G..........................................................................................................  720
QJR94401.1  601   .............G..........................................................................................................  720
QJF76462.1  601   .............G..........................................................................................................  720
QJI54026.1  601   ........................................................................................................................  720
QKG87302.1  601   .............G..........................................................................................................  720
QIV65000.1  601   .............G..........................................................................................................  720
QLA48134.1  601   XXXXXXXXXXX..G...XXXX................XX...............XXXXX.............................................................  720
QKE11591.1  601   .............G.........................................................................................XXXXXXXXXXXXXXXXX  720
QKE11799.1  601   .............G..................................................................................XXXXXXXXXXXXXXXXXXXXXXXX  720
QKG90758.1  601   .............G..........................................................................................................  720
QKR84613.1  601   .............G..........................................................................................................  720
QKV40763.1  601   .............G..........................................................................................................  720
QJR92997.1  601   .............G..........................................................................................................  720
QJR93729.1  601   .............G..........................................................................................................  720
QKV38580.1  601   ........................................................................................................................  720
QJR93825.1  593   .............G..........................................................................................................  712
QKR85561.1  601   .............G..........................................................................................................  720
QIS30355.1  601   ........................................................................................................................  720
QKV37764.1  601   .............G..........................................................................................................  720
QJE39062.1  601   ........................................................................................................................  720
QKV07387.1  601   ........................................................................................................................  720
QJR93165.1  601   .............G..........................................................................................................  720
QJR92229.1  601   ........................................................................................................................  720
QKG88454.1  601   .............G..........................................................................................................  720
QJR91101.1  601   .............G..........................................................................................................  720
QJR91473.1  601   .............G..........................................................................................................  720
QJR88785.1  601   .............G..........................................................................................................  720
QJA17048.1  601   .............G..........................................................................................................  720
QJR86565.1  601   ........................................................................................................................  720
QJR89289.1  601   .............G..........................................................................................................  720
QJR89301.1  601   .............G..........................................................................................................  720
QJY40085.1  601   .............G..........................................................................................................  720
QLC46373.1  601   .............G..........................................................................................................  720
QKE11955.1  601   .............G............................................................XXXXXXXXX................XXXXXXXXXXXXXXXXXXXXX  720
QJQ83440.1  601   .............G..........................................................................................................  720
QJE39145.1  601   ........................................................................................................................  720
QJR91401.1  601   .............G..........................................................................................................  720
QJR90705.1  601   .............G..........................................................................................................  720
QKR85333.1  601   .............G..........................................................................................................  720
QKR84577.1  601   .............G..........................................................................................................  720
QKU31169.1  601   ........................................................................................................................  720
QKV38689.1  601   ........................................................................................................................  720
QJR91209.1  601   ........................................................................................................................  720
QLC46217.1  601   .............G..........................................................................................................  720
QLC46901.1  601   .............G..........................................................................................................  720
QLA48015.1  601   .............G..........................................................................................................  720
QJR93813.1  601   ........................................................................................................................  720
QJA16940.1  601   .............G..........................................................................................................  720
QJR93585.1  601   ........................................................................................................................  720
QKV42562.1  601   ................................X.......................................................................................  720
QJR91653.1  601   ........................................................................................................................  720
QJR90669.1  601   .............G..........................................................................................................  720
QJR94077.1  601   .............G..........................................................................................................  720
QJR89541.1  601   ........................................................................................................................  720
QJR93405.1  601   .............G..........................................................................................................  720
QKR84853.1  601   .............G..........................................................................................................  720
QJI53991.1  601   .............G..........................................................................................................  720
QJR93885.1  601   ........................................................................................................................  720
QJR91593.1  601   ........................................................................................................................  720
QJR89829.1  601   ........................................................................................................................  720
QIQ08820.1  601   .............G..........................................................................................................  720
QJR91761.1  601   ........................................................................................................................  720
QJS57279.1  601   .............G..........................................................................................................  720
QJR95085.1  601   ........................................................................................................................  720
```
